# Combining a transosseous cerclage wire after patellar tendon reattachment to treat patella distal pole fracture did not improve functional outcome

**DOI:** 10.1038/s41598-022-13641-z

**Published:** 2022-06-10

**Authors:** Li-Yang Kuo, Chun-Yu Chen, Kai-Cheng Lin

**Affiliations:** 1 Department of Orthopaedics, Kaohsiung Veterans General Hospital, 386 Ta-Chung 1st Road, Kaohsiung City, Taiwan; 2 Department of Occupational Therapy, Shu-Zen Junior College of Medicine and Management, Kaohsiung City, Taiwan; 3 Department of Biomedical Engineering, I-Shou University, Kaohsiung City, Taiwan

**Keywords:** Trauma, Bone, Ligaments, Muscle, Tendons

## Abstract

This study aims to investigate whether an augmented wire in the treatment of patella distal pole fracture could improve knee range of motion (ROM) and radiographic features. Thirty-five consecutive patients with patellar distal pole fracture were analyzed from January 2014 to July 2019. The treatment is divided into two groups according to the presence or absence of augmented wire. Knee ROM, bone union, extension lag, and patellar height were compared between these two groups as the clinical and radiological outcomes. There was no significant difference in mean knee ROM (110° vs. 108°, *p* = 0.79), proportion of patella baja or bone union. More extension lag was noted in the augmentation group (5/20, 25%) than in the tendon reattachment group (1/15, 6.7%) with no statistically significant difference. In the augmentation group, four cases (20%) would need to remove the fixator due to irritation or broken hardware. Maintaining the patella length by preserving the distal pole and repairing the torn retinaculum allowed early motion to avoid knee stiffness safely without augmentation wire, which doesn’t improve knee ROM. The patellar tendon reattachment alone could achieve a great recovery and prevent the need for a second surgery due to broken wire or irritation.

## Introduction

Patella, the linkage between the quadriceps tendon and tibia tuberosity, acted as one of the essential roles in the extensor mechanism of the knee. The fracture pattern can classify the patellar fractures as transverse, stellate, or vertical. Furthermore, it can be classified as osteochondral and pole fracture based on the separated site. Depending on the type of fracture, different surgical fixations should be applied. A large fragment can be fixed with a screw, and a simple transverse fracture requires the tension band wire technique^1^. However, comminuted fractures require cerclage wire around the entire patella to avoid disrupted fragmentation. A very distal pole pattern with a too-small fragment instructs patellar tendon reattachment. The goals of surgical treatment are to restore the functional integrity and strength of the extensor mechanism, maximize the articular congruity, and preserve the patella maximally. Among all the patella fractures, distal patellar pole fracture accounts for 9.3–22.4%^2^. The avulsed patellar distal pole fragment cannot be stabilized like the case with mid-patellar fracture, which could be securely hold by using a tension band wire, plate, or screw. Therefore, how to restore the knee extensor mechanism without a rigid instrument and maintain the patella height to achieve a better prognosis is the challenge of treating the avulsed patella fracture. In literature, there are multiple solutions that have been published other than partial patellectomy, which is the traditional surgical procedure to re-establish the extensor mechanism. Additionally, circumferential wiring is an intuitive procedure but has the risk of strangling blood vessels around the patella^3,4^. Yang and Byun reported separate vertical wiring techniques and several modifications^5–7^. Furthermore, a basket plate designed for osteosynthesis of the distal patellar pole was also proven to have a good outcome on treating patellar distal pole fracture^8,9^.

When the knee is flexed, the simple patellar tendon reattachment only relies on the strength of the suture itself to connect the quadriceps (proximal pole) and the patellar tendon (distal pole). Moreover, the simple patellar tendon reattachment could not shift the tension force into compression force like tension band wire. Thus, previous studies had suggested using plaster for knee immobilization during the postoperative period to avoid displacements caused by the stretch generated by knee flexion^10,11^. However, prolonged knee immobilization without motion is likely to cause joint stiffness^12^. Adding a transosseous patellotibial cerclage had been suggested to increase the stability of the osteosynthetic construct^13^, which has the benefit of achieving early motion, weight-bearing, and brace-free ambulation^14,15^. Based on our clinical experience, the added device could easily cause knee irritation^16,17^. Thus, the patients would usually request a second surgery to remove the wire. There was still no consensus on whether treating patellar distal pole fracture would have profited from adding extra augmentation suture/wire. The aim of this study is to compare the final clinical outcomes and radiographic features of the following two groups: (1) patellar tendon reattachment only and (2) tendon reattachment with augmentation wire on treating patellar distal pole fracture. We hypothesized that the two groups had no difference.

## Materials and methods

The study included patients who were recruited between January 2014 to July 2019 from our department’s prospective recorded database. The Institutional Review Board of Kaohsiung Veterans General Hospital approved this study. This study was conducted in accordance with the Declaration of Helsinki and its later amendments. All consecutive patients diagnosed with distal pole fracture of the patella were included. The distal pole fracture was defined by two orthopaedic trauma specialists who have confirmed the inability to fix it by tension band wire. All included patients had received postoperative follow-up for at least one year. The patients were excluded if any of the following were present: (1) Ipsilateral knee that had received surgery before, (2) patient who had or previously had trauma-induced limited motion such as contracture or lag before this fracture episode, (3) revision surgery of the patella fracture and (4) being in bedridden status or having the deficient activity of daily living. On the basis of the above criteria, 38 patients (29 were females) with an average age of 62.5 years (range: 23–85) were enrolled. Of these, two cases were open fractures. All the distal patellar fractures did not involve articular surfaces due to minimal distal fragment. Moreover, the surgeon also reconfirmed that none of the patients had fractures affecting the joint surface during surgery.

### Surgical technique

**Figure 1:**
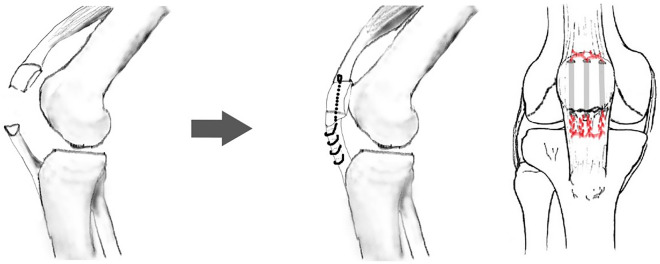
Without sacrificing distal bony fragments, two No.5 Ethibond sutures have been woven through the inferior patella pole and patellar tendon. The four ends of the suture were passed through the drilled tunnel and tied firmly over the intact edge of the patella upper pole.

**Figure 2:**
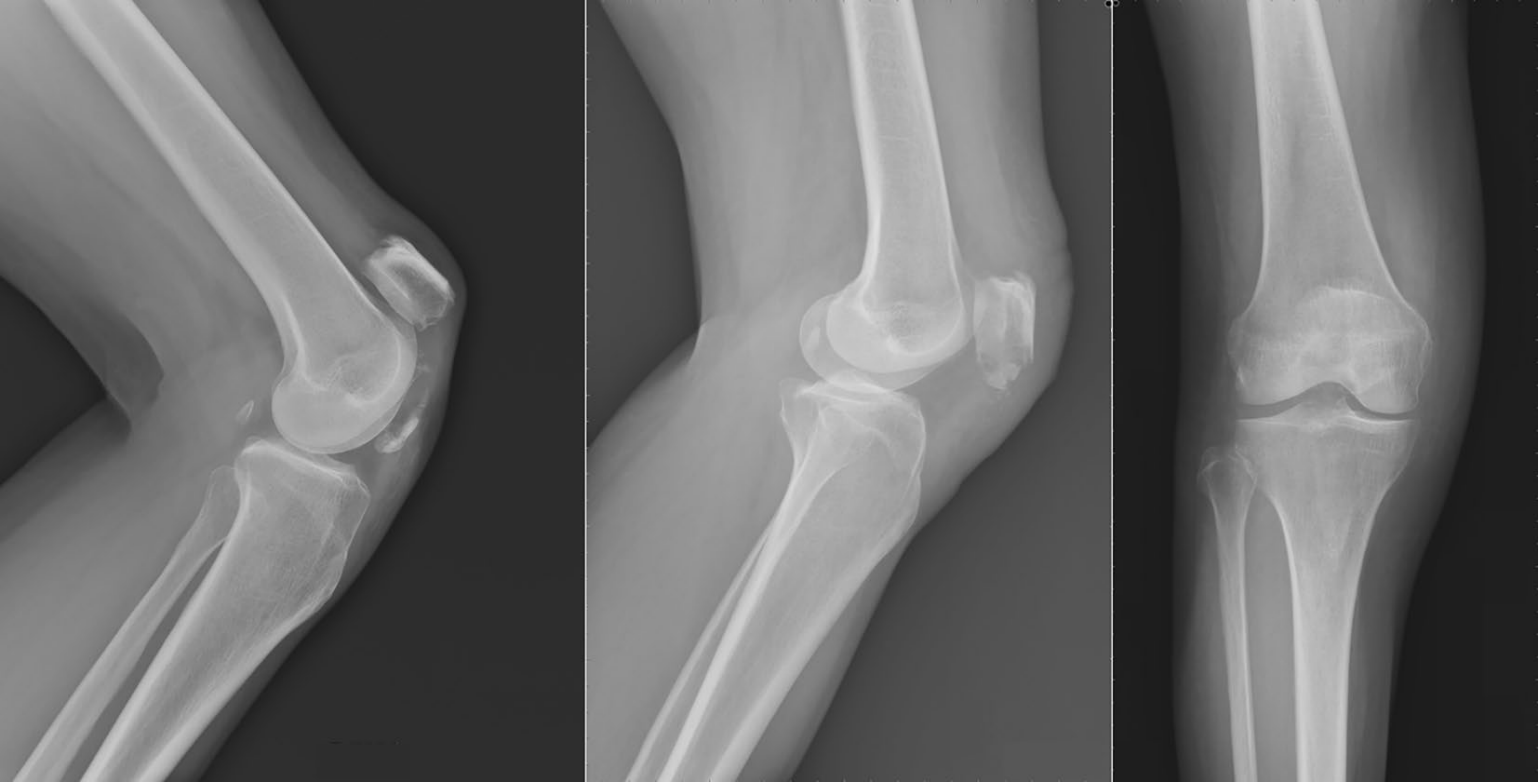
A patellar distal pole fracture was treated with patellar tendon reattachment due to unable to perform the tension band wire fixation.

**Figure 3:**
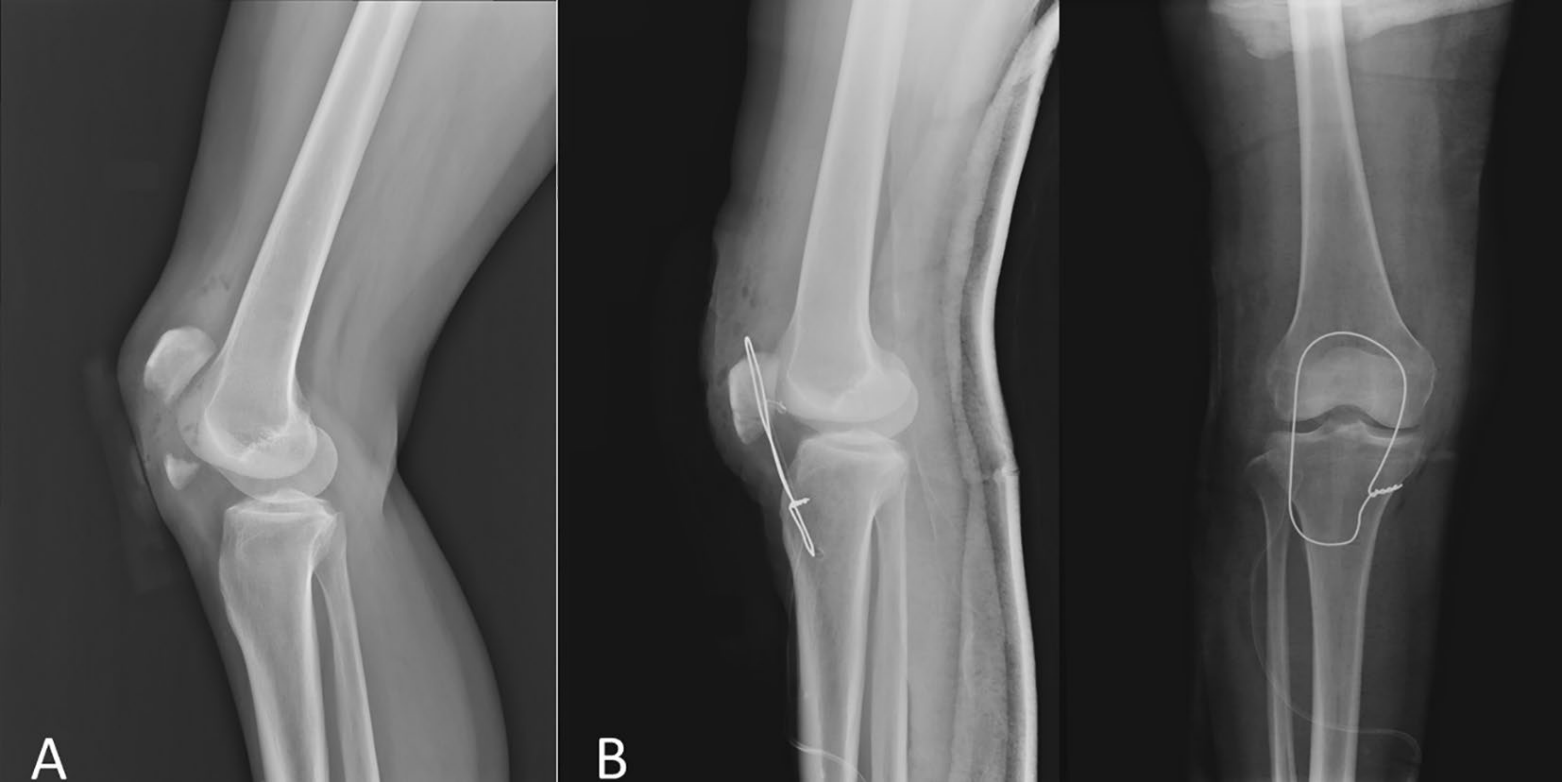
A patellar distal pole fracture was treated with patellar tendon reattachment, and the wire was passed transversely through both the tibial tuberosity and upper patella as a loop for augmentation.

Because of the inability to perform tension band wire fixation, all the patella distal pole fractures were treated with patellar tendon reattachment. Without sacrificing distal bony fragments, obtain the contact of the fractured raw surface during operation. Patella was exposed through a midline longitudinal approach, and the retinaculum was carefully preserved through subcutaneous dissection. After the hematoma was evacuated, the fracture site was exposed. The distal patellar pole fragment was preserved as much as we can unless it was too comminuted to have the risk of becoming loose bodies in the joint or has already become free bony fragment as the devitalized debris. The capsulotomy was not performed because the articular surface was not involved so that it is not required to confirm the reduction of the articulation. Therefore, most of the patella bone blood perfusion was not compromised. Two No. 5 Ethibond sutures used for repair were woven through the medial and lateral halves of the inferior patella pole and patellar tendon with the Krackow technique (Fig. 1). Kirschner wire (2.0 mm or 2.5 mm) was used to drill three longitudinal tunnels through the upper part of the patella from the fracture surface. The four ends of the Ethibond suture were passed through the drilled tunnel and tied firmly over the intact edge of the patella upper pole with the knee in full extension (Fig. 2). After that, No. 1 absorbable Vicryl suture was used to repair the torn medial and lateral retinaculum. The wire was passed transversely through both the tibial tuberosity and the upper patella as a loop for the group with augmentation wire use. Then, the wire was twisted and tightened, while the knee was kept at 90° flexion to resist the power that would make the reattachment separate (Fig. 3).

### Postoperative protocol

All the injured knees were placed in the knee braces, which kept the knee at full extension for two to three days. The patients were encouraged to begin partial weight-bearing ambulation immediately. Once the patient could tolerate the wound pain, gently passive knee motion but less than 30° started. At 10–14 days postoperatively, the passive motion range would gradually increase to at least 90° at week 4 once the wound healed. During the second month after surgery, the enhanced passive motion continued, and active range motion was allowed for muscle training.

### Outcome measurement

The patients enrolled were followed up for at least one year at a single institute, level I trauma center. The range of motion (ROM) of the injured knee and the existence of extension lag were examined in each clinical evaluation. The final assessment at the last outpatient clinic follow-up was presented in this study. The fragment size was evaluated by measuring the proportion of distal pole length to the preserved intact upper patella length in the sagittal plane. Furthermore, the following parameters were measured as the radiographic outcomes: union status, patella height, and loss of reduction. The non-union was defined as no callus formation or no diminished fracture gap after nine months. The patella height was evaluated through the standard knee lateral view on a radiograph. To assess the patella height, we used the Caton–Deschamps ratio^18^. Patella baja was defined when the ratio was < 0.6, whereas the patella alta was determined when the ratio was > 1.3. Loss of reduction was established as the distal pole fragment was displaced on the follow-up radiographs at postoperative two weeks.

### Statistical analysis

The Shapiro–Wilk test was used to examine the presenting values to verify if the parameters belong to normal distribution. The Mann–Whitney U test was used to compare between mean values of the tendon reattachment and augmentation groups. Fisher’s exact test was used to calculate the significance of the difference in outcomes with ratios (patella height, bone union rate, extension lag ratio and distal pole fragment size) of these two groups. The level of significance was set at *p* < 0.05.

### Informed consent

Informed consent was obtained from all individual participants included in the study.

## Results

Three cases receiving revision surgery due to loss of reduction were excluded from the final analysis: one case was in the tendon reattachment without an augmented wiring group, and the other two cases were in the augmentation group. Out of the remaining 35 patients with patella distal pole fracture, 27 were female patients (77.14%). Fifteen patients underwent patellar tendon reattachment and were considered the ‘tendon reattachment group,’ and the other 20 patients received the same patellar tendon reattachment plus augmentation with wiring and were considered the ‘augmentation group’.

Table 1 shows the demographic data for both groups. No significant difference was observed in the open fracture ratio (*p* = 1.0) and fragment size (*p* = 0.52). Regarding the primary outcome measure (Table 2), the mean knee ROM for the tendon reattachment group and the augmentation group was 110.0° and 108.0°, respectively (*p* = 0.79). More extension lag was noted in the augmentation group (5/20, 25%) than in the tendon reattachment group (1/15, 6.7%), but no statistically significant difference was noted (*p* = 0.2). Three patients in each group had a visible gap over the fracture site on radiograph over 12 months, and those cases were defined as non-union. Concerning patellar height, there seems to be a trend in the augmentation group that more cases will show baja, but the difference still did not meet the statistical significance (*p* = 0.067). Because of the broken wire or irritation, only four cases in the augmentation group inquired about removal surgery.

**Table 1:** Descriptive table of demographic variables.

	Tendon reattachment group (n = 15)				Augmentation group (n = 20)				*p*-value
Female	12 (80%)				15 (75.0%)				1.00*
Open fracture	1 (6.7%)				1 (5.0%)				1.00*

	Mean	Min	Max	SD	Mean	Min	Max	SD	
Age, years	58.67	23	85	14.63	61.05	24	85	15.46	0.59^+^
Fragment size, percent	27.53	9	41	7.80	27.55	16	43	7.57	0.52^+^

*Fisher’s exact test.

^**+**^Mann–Whitney U test.

**Table 2:** Descriptive table of outcome variables.

	Tendon reattachment group (n = 15)				Augmentation group (n = 20)				*p*-value
	Mean	Min	Max	SD	Mean	Min	Max	SD	
ROM, degrees	110	80	140	18.9	108	60	140	23.8	0.79^+^
Extension lag	1 (6.7%)				5 (25%)				0.20*
Bone union	12 (80.0%)				17 (85.0%)				1.0*
**Patella height**									0.067*
Normal	14 (93.3%)				14 (70.0%)				
Baja	0 (0%)				5 (26.3%)				
Alta	1 (6.7%)				1 (5.0%)				
Implant removal	0 (0%)				4 (20%)				0.11*

*ROM* range of motion.

^+^Mann–Whitney *U* test.

*Fisher’s exact test.

## Discussion

Commonly, patella fracture was treated by open reduction and internal fixation (ORIF) with tension band wire, which transforms the tension force into compression force for maintaining compaction and stability. However, the ORIF with tension band wire was not proper for distal pole patella fracture because of the minor or comminuted fragments; thus, the previous surgeons have sought a different way of treating distal pole patella fracture^10,19^. In the past, much research had proved the effectiveness of partial patellectomy in re-establishing the extensor mechanism at treating patella distal pole fracture^10,11^. In the last two decades, many surgeons considered patella distal pole fracture as an avulsion fracture, and many pole reservation methods were published^5–9,20^. Kastelec et al. indicated that pole reservation had advantages such as early weight-bearing and immediate mobilization compared to the pole resection method^9^.

A retrospective study by West et al. evaluated a method that adds a relaxing cerclage suture around the patella and protects the torn patellar or quadriceps tendon after repair. They found this protecting device was strong enough to safely permit early motion, weight-bearing, and brace-free ambulation after surgery^15^. Furthermore, they listed 12 studies between 1987 and 2006 supporting the effectiveness of the augmentation device in allowing early joint motion after the operation. However, in half of the above studies, secondary surgery for device removal had been mentioned. Many studies have supported the use of augmentation devices, either wire, suture, graft, or synthetic Dacron vascular graft to protect the non-healing repaired site of the patellar or quadriceps tendon. In our literature review about treating patellar distal pole fracture, these studies lacked control groups and thus had less persuasive point to indicate that adding extra augmentation device is superior to only simple repair.

Early motion after stable fixation of patellar fracture is generally considered the best way to avoid knee stiffness, which is believed to be one of the most challenging complications after patellar surgery. Because the patellar distal pole fracture is recognized equivalent to patellar tendon avulsion, it is worth discussing whether it is necessary to add augmentation wire to drop out the separating forces encountered during knee motion after patellar tendon reattachment. In this study, we compared 35 patella distal pole fracture cases, and the results revealed that there was no difference in knee ROM after surgery with or without the augmentation wiring after long-term follow-up (> 12 months). Furthermore, no difference was noted at the bone union or patella position. The augmentation group had more cases of extension lag but had not reached statistical difference. The proportions of loss of reduction were similar in the two groups; however, the augmentation group had more cases (two versus one).

The arteries nourish the patella in two ways. The first through running anterior to the patella and entering the bone via the vascular foramina surrounding the patella border. The second way was from the infrapatellar anastomosis behind the patellar tendon, which was formed by the transverse infrapatellar branches of the inferior genicular arteries^4,21^. Repairing the retinaculum and preserving the inferior pole retained the blood supply, leading to a better biochemical environment for bone and tendon to heal. Furthermore, distal pole preservation preserves the patellar length and maintains the relative position of the patella and patellar tendon. It could release the tension in the patellar tendon insertion on the distal pole, which was better for bone-to-bone and bone-to-tendon healing. In previous research, it was also mentioned that the infrapatellar contracture syndrome that induced patella baja easily happened when the patellar length was changed^12^. Matej Kastelec et al. also found a better functional outcome for patients with preserved patellar distal pole than pole resection on treating patella distal pole fracture^8^. Our study’s results revealed that well-executed patellar tendon reattachment can achieve good therapeutic results, and augmentation wiring does not appear to be of significant benefit.

In some situations, the remaining patella and the fragmentary distal pole are far apart on the initial radiograph, indicating a severe rupture of the retinaculum and capsule, and the surgeons then attempt passively flex the knee that just following patellar tendon reattachment, it is recommended to add the augmented wire if the gap expanded. Except for the above situations, the augmented wire is unnecessary to avoid irritation. Gunasekaran Kumar et al. disclosed that one third of their patients would require secondary surgery due to implant-related symptoms^22^. In this study, most patients received a single surgery without later implant removal. However, those cases that needed secondary surgery for implant removal belonged to the augmentation group totally, which accounts for 20%. In our cases, removing implants was due to broken wire (three cases) and irritation (one case).

There are several limitations to this study. First, the number of patients enrolled was relatively small owing to an unusual injury pattern. Second, several surgeons in our institute randomly treated these patients, and every surgeon had their indication of whether to add the augmentation wire or not. Selection bias may therefore exist. However, the demographic variables such as fragment size did not appear a difference between the two groups. Last, this study could not accurately indicate patient satisfaction since it lacked a comparison of daily activity level and knee function score.

## Conclusion

This study stated that adding an augmentation device to treat patella distal pole fracture did not improve knee ROM after long-term follow-up. The crucial points are to maintain the patella length by preserving the distal pole and repairing the torn retinaculum firmly, which allowed an early motion to avoid knee stiffness more safely. In this way, only patellar tendon reattachment alone could achieve a great recovery and prevent the need for a second surgery due to broken wire or irritation.

## Data Availability

The datasets used and/or analyzed during the current study available from the corresponding author on reasonable request.
